# Mobile continuous-flow isotope-ratio mass spectrometer system for automated measurements of N_2_ and N_2_O fluxes in fertilized cropping systems

**DOI:** 10.1038/s41598-019-47451-7

**Published:** 2019-07-31

**Authors:** Daniel I. Warner, Clemens Scheer, Johannes Friedl, David W. Rowlings, Christian Brunk, Peter R. Grace

**Affiliations:** 1 Institute of Future Environments, Queensland University of Technology, Brisbane, QLD 4000 Australia; 2 Institute for Meteorology and Climate Research- Atmospheric Environmental Research (IMK-IFU), Karlsruhe Institute of Technology (KIT), Garmisch-Partenkirchen, Germany

**Keywords:** Element cycles, Environmental monitoring

## Abstract

The use of synthetic N fertilizers has grown exponentially over the last century, with severe environmental consequences. Most of the reactive N will ultimately be removed by denitrification, but estimates of denitrification are highly uncertain due to methodical constraints of existing methods. Here we present a novel, mobile isotope ratio mass spectrometer system (Field-IRMS) for *in-situ* quantification of N_2_ and N_2_O fluxes from fertilized cropping systems. The system was tested in a sugarcane field continuously monitoring N_2_ and N_2_O fluxes for 7 days following fertilization using a fully automated measuring cycle. The detection limit of the Field-IRMS proved to be highly sensitive for N_2_ (54 g ha^−1^ day^−1^) and N_2_O (0.25 g ha^−1^ day^−1^) emissions. The main product of denitrification was N_2_ with total denitrification losses of up to 1.3 kg N ha^−1^ day^−1^. These losses demonstrate sugarcane systems in Australia are a hotspot for denitrification where high emissions of N_2_O and N_2_ can be expected. The new Field-IRMS allows for the direct and highly sensitive detection of N_2_ and N_2_O fluxes in real time at a high temporal resolution, which will help to improve our quantitative understanding of denitrification in fertilized cropping systems.

## Introduction

In the last 50 years, the use of synthetic nitrogen (N) fertilizers, cultivation of leguminous crops, and fossil fuel combustion have more than doubled the input of reactive nitrogen (N_r_ – all nitrogen species other than dinitrogen N_2_) into the environment. As a result, the global N cycle is even more severely altered by human activity than the global carbon (C) cycle, and excess N pollution has been identified as one of the three global environmental issues whose ‘planetary boundary’ has been surpassed^1^. Once an N atom is in a reactive form (N_r_), it cascades through different eco-system compartments, contributing to a number of environmental problems^2^. Most of the anthropogenic N_r_ will be removed by denitrification, the reduction of oxidized forms of inorganic N to N_2_O and, ultimately, inert N_2_. However, estimates of terrestrial sinks for N_r_ are highly uncertain and the magnitude of total denitrification losses (N_2_ + N_2_O) is virtually unknown for the vast majority of agricultural soils. While there is increasing research investigating the soil-atmosphere exchange of N_2_O in fertilized agro-ecosystems, information on N_2_ emissions and related N_2_:N_2_O partitioning data is still scarce^3^. The main reasons for this uncertainty are that denitrification exhibits a very high spatial and temporal variability, but foremost that it is extremely difficult to measure N_2_ emissions against the huge atmospheric N_2_ background^4^.

Currently, there are only two main approaches for the direct quantification of denitrification N gas formation including N_2_ and N_2_O in the field: (1) the Acetylene Inhibition Technique (AIT) and (2) the ^15^N-gas flux method. The AIT is historically the most widely applied method to quantify denitrification in the field due to its relatively simple and inexpensive application. However, in recent years it has been shown that the AIT leads to a systematic and irreproducible underestimation of denitrification rates due a number of limitations^3–6^, which preclude reliable estimates of total denitrification losses^7^. The shortcomings of the AIT therefore demand the use of alternative methods when quantifying denitrification losses from agro-ecosystems. The ^15^N-gas flux method avoids the problems of the AIT, but requires expensive equipment as well as the costly application of highly ^15^N enriched fertilizer. ^15^N is added as highly enriched (20–80 atom %) fertilizer to a designated plot of soil which is then enclosed with a chamber prior to gas sampling. N_2_ and N_2_O gas production following denitrification is measured by quantifying the increase in ^15^N-labelled gases in the headspace using Isotope Ratio Mass Spectrometry (IRMS)^8^. The major challenges of the ^15^N tracing technique are (1) achieving and assessing uniform distribution of the isotopically labelled N in the soil (2) artificial stimulation of denitrification by the added tracer (3) high detection limits for N_2_ fluxes due to analytical limitations in the *m/z* 30 measurements with the IRMS. The uniform distribution of ^15^N in the soil nitrate (NO_3_^−^) pool is usually achieved by applying ^15^N enriched fertilizers with water, resulting in increased soil moisture. Under field conditions it is unlikely to achieve homogeneity of the added ^15^N tracer with the soil NO_3_^−^ pool, but it has been shown that accurate estimates of gaseous N losses can be made without uniform distribution of ^15^N in the soil when large amounts of highly enriched N fertilizer are applied^9^. Consequently, the ^15^N gas flux method is most applicable for fertilized cropping systems where large amounts of enriched N fertilizer with water can be applied and high fluxes of N_2_ and N_2_O can be expected.

Another constraint for field measurements of denitrification is the high temporal (diurnal, daily and seasonal) variability of N gas emissions that compromise flux estimates if not carried out with an adequate frequency. For N_2_O fluxes it has been shown that daily sampling is required to achieve annual N_2_O fluxes within 10% of the best estimate, while weekly to monthly sampling in cropping systems with highly episodic emission events can result in an overestimation of 256% and 935%, respectively^10^. To address this problem more and more studies are using automated static chamber systems that can capture highly episodic emissions, and the characteristic diurnality in emissions, by multiple sampling events over any 24-hour period. Such “high-frequency” measurements of N_2_O have significantly improved N_2_O flux estimates. However, such instrumentation has not been available for both N_2_ and N_2_O measurements.

Here we present a novel, mobile isotope ratio mass spectrometer (Field-IRMS) coupled to a fully automated chamber system, measuring N_2_ and N_2_O fluxes in real time at a sub-daily resolution. The Field-IRMS is housed in an air-conditioned trailer and can be transported to the desired field location. We tested the performance of the Field-IRMS investigating the effect of two different fertilizer rates on the magnitude and N_2_:N_2_O partitioning of denitrification losses from a sugarcane field in subtropical Australia.

## Methods

### Field-based IRMS system

The new Field-IRMS was developed by the Queensland University of Technology (QUT) in collaboration with Sercon (Sercon, Crewe, UK) and is housed in an air-conditioned trailer which can be transported to the desired field location. The system uses a standard continuous flow IRMS coupled to a fully automated chamber system, measuring N_2_ and N_2_O fluxes in real time at a sub-daily resolution. The measuring principle is based on the ^15^N gas flux method and uses highly ^15^N enriched (20–80 atom % ^15^N) fertilizer added to the soil which is then enclosed with a chamber for a designated time period. N_2_ and N_2_O gas production from denitrification is measured by quantifying the increase in ^15^N-labelled gases in the chamber atmosphere using IRMS^8^.

**Figure 1:**
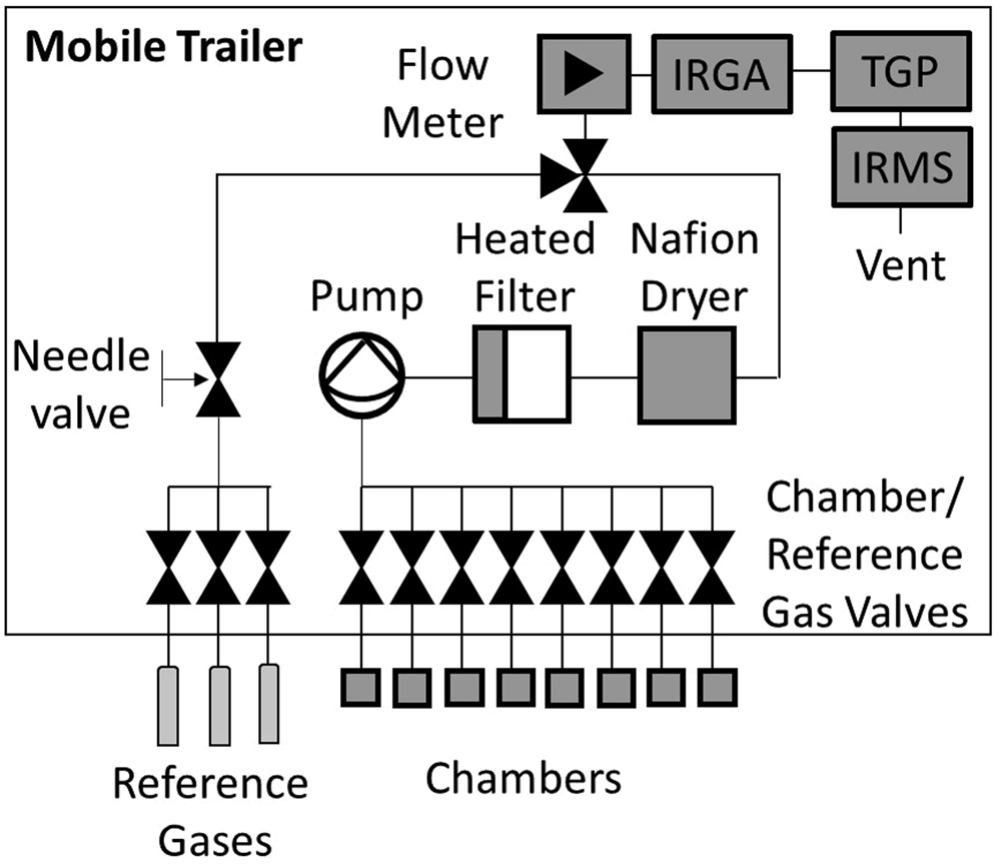
The setup of the field-based IRMS system: Automated chambers and the sampling and reference gas injection unit, the infra-red gas analyser (IRGA), the trace-gas preparation unit (TGP) and the IRMS.

The new Field-IRMS comprises three main parts: The automated chambers, the sampling unit, and a Sercon Continuous Flow 20–22 IRMS with a custom built trace gas preparation unit (TGP) (Fig. 1).

### Automated chambers

The system utilises the static closed chamber technique (non-steady-state, non-through flow) to capture N_2_ and N_2_O fluxes from soils. It consists of eight automated acrylic static chambers (500 mm × 500 mm × 150 mm) that are fixed on stainless steel bases inserted permanently into the soil to a depth of 100 mm and sealed air-tight during sampling by two lids that open and close via pneumatic actuators. It was assumed that molecular diffusion created homogeneous gas concentrations in the chamber headspace at sampling and no fan to mix the air inside the chamber was installed. To limit the temperature increase in the chambers during the closure times the acrylic panels on the side of the chambers were tinted while the panels on the two lids of each chamber were covered with reflective foam insulation. Each chamber is connected to a sampling control system by a non-reactive Teflon coated sample line and two pneumatic air lines (for lid open and shut operation).

### Sampling unit

The sampling system houses the sample pump, sample valves, reference gas valves, sample flow meter, an infra-red CO_2_ analyser for the measurement of soil CO_2_ fluxes (LI-840, LI-COR Biosciences, US), and a filtering system (Fig. 1). It is designed to extract sample gas from 8 chamber and three reference gas locations, which can be injected into the TGP at precise times during the sampling cycle. Each chamber and reference gas cylinder is connected to a single solenoid valve (Bürkert, Ingelfingen, Germany) arranged in arrays of eight and three, respectively. When either of the chamber valves are opened a diaphragm pump (JAVAC, Australia), provides constant air flow (200 mL min^−1^) to the TGP from the chamber selected. For reference gas injection a three port valve is used to isolate the chamber valves from the reference gas valve stream. An adjustable flow meter (Key Instruments, Hatfield, USA) ensures constant flow from the sampled chamber headspace. A heated 0.1 micron coalescing filter and Nafion™ dryer filter system (Perma Pure, Lakewood, USA) removes particulate and water vapour from the gas sample.

### Trace gas preparation unit

**Figure 2:**
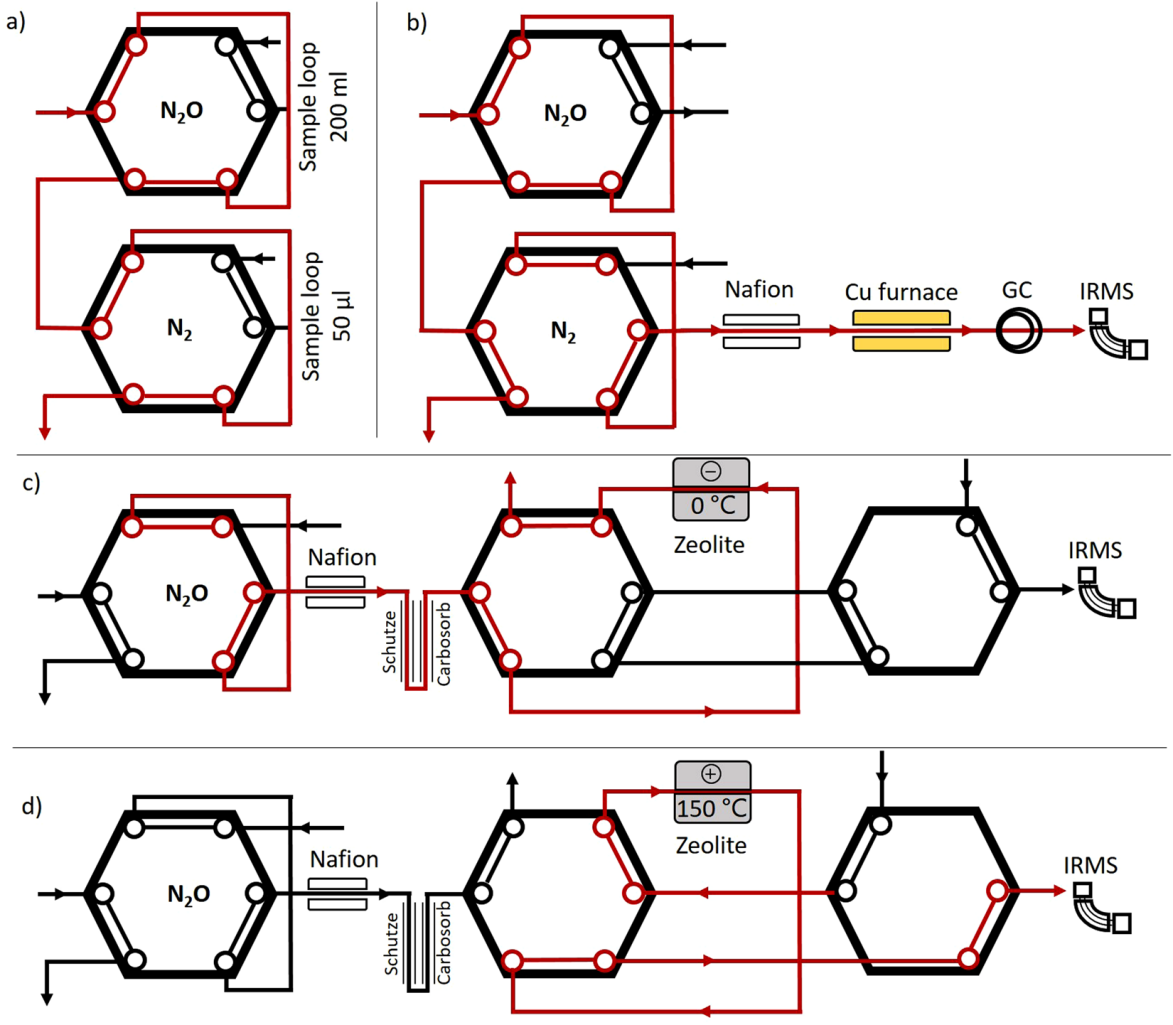
The trace gas preparation unit, with (**a**) N_2_ and N_2_O sample loop in filling position (**b**) the N_2_ sample loop switched to the N_2_ analysis path (**c**) the N_2_O sample loop switched the N_2_O path, trapping N_2_O in the Zeolite trap, and (**d**) the release of N_2_O to the IRMS, after the heating of the Zeolite trap.

The custom built TGP works on the same principle as the standard Sercon Cryoprep trace gas module (Sercon, Crewe, UK), which isolates and purifies discreet amounts of trace gases to be transferred to the IRMS for isotopic analysis. The installation of two six-port Valco valves (VICI-Valco Instruments, Houston, USA) allows the incoming sample flow to fill a 200 mL N_2_O sample loop and 50 µL N_2_ sample loop (Fig. 2a). Once the sample loops are filled the sample is injected into the helium carrier flow (20 ml min^−1^) of the TGP, along two separate pathways: an N_2_ + N_2_O path and an N_2_O path.

### N_2_ + N_2_O path

Along the N_2_ + N_2_O path, the sample is passed through a Nafion™ tube, a reduction oven and a GC column (Fig. 2b). The Nafion™ tubes remove any remaining water still present in the sample. The reduction oven of reduced copper heated to 600 °C removes O_2_ from the sample. This is essential because any O_2_ left in the sample will interfere with the isotopic analysis due to the production of NO and thus *m/z* 30 in the ion source of the IRMS. Moreover, the copper furnace reduces any NO or N_2_O to N_2_, which will be subsequently detected as N_2_. Hence, it needs to be noted that the N_2_ + N_2_O path will also contain any NO emitted from the soil which could result in an overestimation of the total N_2_ fluxes. A molsieve (5 Å) packed GC column with a diameter of ¼” and length of 20 cm separates N_2_ from any remaining O_2_.

### N_2_O path

On the N_2_O path, a Nafion™ tube, CO to CO_2_ converter, CO_2_ scrubber, and zeolite N_2_O trapping system are installed. Once the N_2_O sample is transferred to the helium carrier flow it first passes through the nafion tube to remove any residual water. The sample then passes through a two-part carbon scrubber: any CO in the sample is converted to CO_2_ using a Schuetze reagent column, followed by a Carbosorb^TM^ packed column removing CO_2_ (Fig. 2c,d).

In commercially available trace gas preparation units, the isolation and concentration of N_2_O is achieved using a liquid N (LN) trap. This requires a constant supply of LN which is not practical at remote field sites. The LN trap has therefore been replaced with a zeolite-based N_2_O trapping system. The sample is passed through a cooled zeolite column for 423 seconds which effectively traps N_2_O. Once the N_2_O is trapped, the zeolite trap is isolated from the helium carrier stream by switching the respective Valco valve. The zeolite trap is then heated for 125 seconds, releasing the trapped N_2_O. The now concentrated N_2_O is then directed back into the helium carrier and transferred to the IRMS. To trap the N_2_O, the zeolite is cooled to near 0 °C by using Peltier CPU coolers (RS Components, Sydney, Australia). Standard cartridge heaters (RS Components, Sydney, Australia) are used to rapidly heat the zeolite column to 150 °C and release the N_2_O from the zeolite trap.

### Automated sampling cycle

The N_2_ and N_2_O fluxes are measured using a fully automated sampling cycle during which four gas samples are taken sequentially from each chamber over a 201.67 min period with a total closure time of 221.83 min. During each of the sampling runs different reference standards are analysed alternating with the samples. The reference standards consisted of ambient air samples and a 1 ppm N_2_O standard, injected from a certified calibration gas cylinder (SupaGas, Beenleigh, QLD, Australia). The ambient air samples were used to provide an isotopic reference for the N_2_ measurements, and the N_2_O calibration standard was used to provide a reference for the N_2_O measurements. More details of the automated sampling cycle are provided in the Supporting Information.

### Flux calculations

Di-nitrogen and the converted N_2_O (N_2_ + N_2_O) are measured as N_2_ in the IRMS, while N_2_O is measured directly as N_2_O. For N_2_O, the ion currents (I) at *m/z* 44, 45 and 46 enabled the calculation of the molecular ratios ^45^R (^45^I/^44^I) and ^46^R (^46^I/^44^I), giving the fraction of N_2_O derived from the NO_3_^−^ pool undergoing denitrification of the enrichment *a*D^11^. The concentration of N_2_O is calculated based on measured (^45^R, ^46^R) and calculated (^47^R, ^48^R) ratios of N_2_O isotopologues in each sample compared to those in of the 1 ppm reference^12^. For N_2_, the I at *m/z* 28, 29 and 30 enabled ^29^R (^29^I/^28^I) and ^30^R (^30^I/^28^I) to be calculated, with differences between ambient and enriched atmospheres expressed as Δ^29^R and Δ^30^R. The flux of N_2_ + N_2_O was calculated using Δ^30^R and *a*D^11,13^, assuming that both N_2_O and N_2_ were produced from a single source pool. Flux rates of N_2_O were calculated from the slope of the linear increase in gas concentration during the closure period. Fluxes of N_2_ were then calculated as the difference between N_2_ + N_2_O and N_2_O fluxes. The coefficient of determination (r^2^) was used for both N_2_ + N_2_O and N_2_O as a quality check for linearity and flux rates were discarded if r^2^ was <0.80 (Fig. S1). Flux rates were corrected for temperature, air pressure and the ratio of cover volume to surface area^14^. For more details, see Supporting Information.

### Field experiment

To test the performance of the novel system under field conditions we investigated the effect of two different fertilizer rates on the magnitude and the partitioning of N_2_ and N_2_O denitrification losses from a sugarcane cropping system in subtropical Australia (24°57′53″S, 152°20′0″E). Fertilizer (K^15^NO_3_ at 60 atom %, Sigma Aldrich) was applied to the chamber bases at a rate of 50 kg N ha^−1^ (50 N) and 100 kg N ha^−1^ (100 N), with four replicates arranged randomly along two sugarcane rows. The fertilizer was dissolved into 1 L of water and applied by hand, to each chamber, to ensure even distribution. Prior to the application of the fertilizer 3 L of water was applied to each chamber to ensure a wet soil profile while minimizing the potential for leaching losses of ^15^N labelled fertilizer. Following the application of the fertilizer, a 5 cm thick layer of sugarcane trash was placed in each chamber and irrigated with a further 6 L of rainwater. Therefore, a total of 10 L irrigation was added to each chamber, which simulated a 50 mm rain event to completely saturate the soil. The addition of the sugarcane trash is consistent with farming practices in the growing region. More details of the experimental site are given in the Supporting Information.

### Soil sampling and analysis

Initial soil samples were taken to 10 cm depth before fertilizer application and after four days from each edge and the centre of each soil frame using a 5 cm soil auger. One day after the final N_2_ and N_2_O flux measurements, final soil samples were taken from the profile in 10 cm increments to a depth of 50 cm.

The soil samples were analysed for total mineral N by extracting the NH_4_^+^ and NO_3_^−^ in a 20 g subsample of soil. The subsamples were mixed in 100 mL of KCl (1:5 solutions) and shaken for one hr before being filtered through Whatman no. 42 filter paper. The KCl extracts were then analysed for N-NH_4_^+^ and N-NO_3_^−^ on an AQ2^+^ discrete analyser (SEAL Analytical WI, USA).

The ^15^N enrichments of the N-NO_3_^−^ and N-NH_4_^+^ was determined for the 0–10 cm samples by the diffusion method^15^. After the diffusion process was complete, the samples were analysed on a Sercon 20–22 IRMS.

### Auxiliary measurements

Soil moisture and temperate was measured at a depth of 10 cm in four of the eight chambers using automatic probes (HOBOnodes, Onset, USA). These probes collected moisture and temperature reading every 5 seconds for the duration of the experiment. Chamber temperature was also recorded in one chamber of each set.

### Statistical analysis

Statistical analyses were conducted with SPSS 22.0 (SPSS Inc., 2013). Treatment effects on N_2_ and N_2_O fluxes were examined by analysis of variance (ANOVA) (*P* < 0.05). Changes over time were analysed using one way repeated measures ANOVA (*P* < 0.05), where time was the repeated factor. Values in the figures represent means ± standard error of the mean.

## Results

### Performance of the Field-IRMS system

The precision of the Field-IRMS for N_2_ analysis was evaluated using the between – batch standard deviation of ambient air samples (n = 126) included in the sampling run over the seven-day experimental period. The detection limit (DL) of N_2_ was calculated based on the standard deviation (SD) of these atmospheric air samples. The SD was 2.85 * 10^−07^ and 8.55 * 10^−07^ for the true mass ratios ^29^R (29/28) and ^30^R (30/28), respectively. The detection limit was calculated using equation 1:

1 $${\rm{DL}}={{\rm{T}}}_{({\rm{n}}-11-\alpha =0.95)}\,\ast \,{\rm{SD}}$$

**w**here T_(n−1,1−α=0.95)_ is the student’s t value at a 95% confidence level at n-1 degrees of freedom and SD is the respective standard deviation. The between batch DL of the Field-IRMS at the 95% confidence interval (n = 126) for ^29^R was 4.70 * 10^−07^ and for ^30^R was 1.41 * 10^−06^. This equates to a method DL (MDL) for N_2_ fluxes of 54 g ha^−1^ day^−1^ of N_2_ based on a NO_3_^− 15^N pool enrichment of 50% and a closure time of 201 minutes.

The precision of the IRMS for N_2_O measurements was determined based on repeated analyses of an N_2_O gas standard (n = 12) with a concentration of 1 ppm at natural abundance injected into the TPG unit. The SD was 3.79 * 10^−04^ and 9.08 * 10^−05^ for the true mass ratios ^45^R (45/44) and ^46^R (46/44), respectively. The SD of the N_2_O concentration was 0.013 ppm, which equates to a DL at a 95% confidence interval of 0.02 ppm (equation 1). The resulting MDL for N_2_O was 0.25 g N_2_O-N ha^−1^ day^−1^.

### Field test

We were able to measure significant fluxes of both N_2_ and N_2_O over the entire seven day field test in the sugarcane cropping system, with a significant accumulation of ^15^N-N_2_ and N_2_O in the chamber headspace over the 201 min measuring time (Fig. S1). For N_2_O a linear increase in the chamber headspace could be confirmed, with r^2^ > 0.9 for the regression of the N_2_O concentration versus time for all measurements. For N_2_ a linear increase in ^15^N-N_2_ over time was confirmed for most measurements with r^2^ > 0.8 for the regression of the ^15^N concentration versus time for 93% of all flux measurements, while 7% of the fluxes were discarded. The enrichment of the soil NO_3_^−^ pool undergoing denitrification derived from ^15^N_2_O (*a*D) and from ^15^N_2_ were not significantly different, except for two days in each treatment (Fig. S2).

Total N_2_ + N_2_O losses over the seven-day monitoring period resulted in 2.10 ± 0.46 kg ha^−1^ and 6.12 ± 0.99 kg ha^−1^ lost for the 50 N and 100 N treatments, respectively (Table 1). N_2_O from denitrification (N_2_O_d_) accounted for 78% (50 N) and 91% (100 N) of total N_2_O emissions. N_2_ was the main product of denitrification in both treatments, with an N_2_/(N_2_ + N_2_O) product ratio of 0.93 and 0.90 of the total in the 50 N and 100 N treatment, respectively. Cumulative N_2_ emissions exceeded N_2_O emissions by a factor of 10 ± 2 in the 50 N treatment and a factor of 17 ± 5 in the 100 N treatment. Of the cumulative denitrification losses (N_2_ + N_2_O) 77% and 80% were fertilizer derived in the 50 N and 100 N treatments, respectively.

**Table 1:** Total N losses and the resulting product ratio of denitrification (N_2_/N_2_ + N_2_O ratio) over the seven day monitoring period.

Treatment	Total N lost	N_2_	N_2_O	N_2_O_d_	Product ratio of denitrification
	kg N − N_2_ + N_2_O ha^−1^	kg N − N_2_ ha^−1^	kg N − N_2_O ha^−1^	kg N − N_2_O_d_ ha^−1^	N_2_/(N_2_ + N_2_O_d_)
50 N	2.10 ± 0.46^a^	1.97 ± 0.44^a^	0.13 ± 0.04^a^	0.10 ± 0.03^a^	0.93 ± 0.02^a^
100 N	6.12 ± 0.99^b^	5.45 ± 0.78^b^	0.67 ± 0.22^a^	0.61 ± 0.2 ^a^	0.90 ± 0.02^a^

Means denoted by a different lowercase letter indicate significant differences (P < 0.05) between treatments.

**Figure 3:**
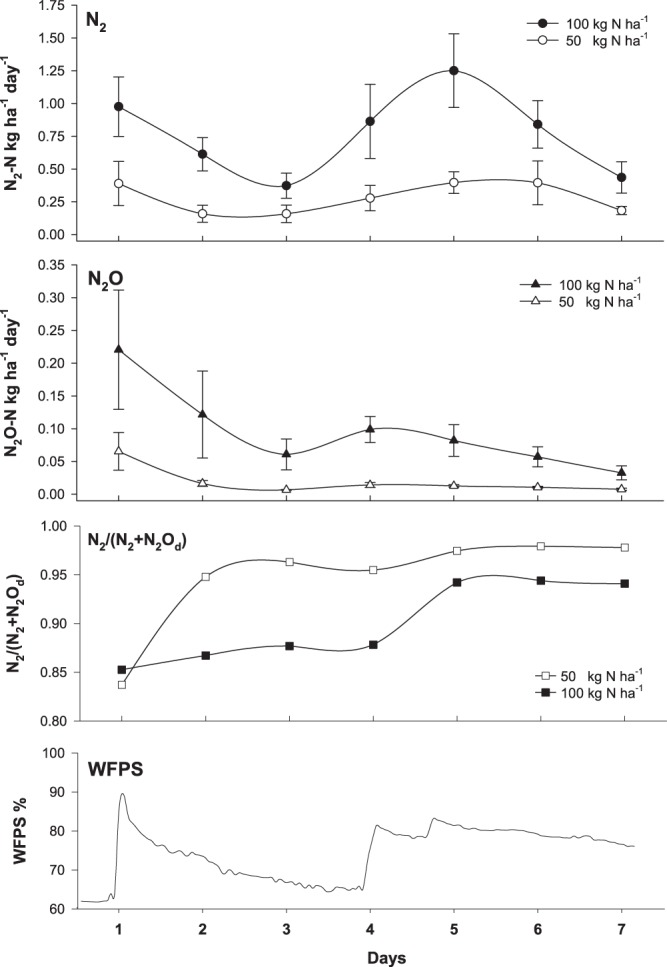
Temporal pattern of N_2_ and N_2_O fluxes (kg-N ha^−1^ day^−1^) and the corresponding product ratio of denitrification (N_2_/N_2_ + N_2_O ratio) over the seven day monitoring period for the two fertilizer application rates of 50 kg-N ha^−1^ and 100 kg-N ha^−1^. The bottom graph shows the water-filled pore space over the monitoring period based hourly averages.

The temporal pattern of mean N_2_ fluxes shows a pulse of N_2_ emissions after fertilization and of irrigation (Fig. 3). On day one, 0.39 ± 0.17 g N_2_-N ha^−1^ day^−1^ and 0.98 kg ± 0.23 N_2_-N ha^−1^ day^−1^ were emitted from the 50 N and 100 N treatment, respectively. Subsequent N_2_ fluxes decreased in both treatments with lowest N_2_ fluxes at day 3 regardless of treatment. A second irrigation event of 25 mm during the night of the 3^rd^ day led to a second peak in N_2_ emissions on day 5, with N_2_ fluxes of 0.40 ± 0.08 N_2_-N ha^−1^ day^−1^ and 1.25 ± 0.28 kg N_2_-N ha^−1^ day^−1^ for the 50 N and 100 N treatment, respectively.

Nitrous oxide emissions were highest on day 1 in both treatments and decreased over the course of the experiment (Fig. 3), exhibiting a small spike on day 4 in both treatments. Nitrous oxide from the 50 N treatment decreased from 65.4 ± 28.70 g N_2_O-N ha^−1^ day^−1^ on day 1 to 7.6 ± 1.64 g N_2_O-N ha^−1^ day^−1^ on day 7. In the 50 N treatment N_2_O emissions peaked on day 1 at 221 ± 9.11 g N_2_O-N ha^−1^ day^−1^ with a subsequent decrease to 32.7 ± 1.06 g N_2_O-N ha^−1^ day^−1^ on day 7.

The temporal pattern of product ratio of denitrification (N_2_/N_2_ + N_2_O) shows N_2_ as the main product of denitrification over the entire course of the experiment in both treatments (Fig. 3). Lowest ratios were observed on day one at 0.84 and 0.85, for the 50 N and 100 N treatment, respectively. In the 50 N treatment, the N_2_/N_2_ + N_2_O increased above 0.90 after day 1 to 0.98 at day 7, while the N_2_/N_2_ + N_2_O ratio of the 100 N treatment remained below 0.90 until day 5, increasing to 0.94 at day 7.

The soil NO_3_^−^ concentration decreased over the first 4 days of the experiment in both treatments to less than 20% of the initially applied fertilizer (Table 2). This rapid decline continued until day 8 with only 7% of the applied NO_3_^−^ detectable in the top 10 cm of the soil. The enrichment of the ^15^NO_3_^−^ pool declined from a theoretical value of 55 atom% ^15^N to values below 20 atom%. The soil NH_4_^+^ pool displayed no significant changes over time but exhibited ^15^N enrichment over the course of the experiment indicating movement of the added ^15^NO_3_^−^ fertilizer into the soil NH_4_^+^ pool.

**Table 2:** Soil mineral nitrogen levels (NO_3_^–^-N, NH_4_^+^ -N), the ^15^N enrichment of the soil mineral nitrogen pools (NO_3_^–^-^15^N, NH_4_^+^-^15^N) and the fraction of the ^15^N labelled nitrate pool undergoing denitrification (*a*D)) at day 1, 4 and 8 of the field experiment.

Measurements	Treatment	Days		
		0	4	8
NO_3_^−^ (kg-N ha^−1^)	50 N	51.9*	9.13 ± 0.55^a^	3.87 ± 1.51^b^
	100 N	101.9*	18.44 ± 7.06^a^	7.73 ± 4.61^ab^
NH_4_^+^ (kg-N ha^−1^)	50 N	8.31	5.51 ± 0.43^a^	6.04 ± 0.79^a^
	100 N		5.84 ± 0.48^a^	6.88 ± 0.47^a^
^15^NO_3_^−^ (atom% ^15^N)	50 N	54.93*	28.77 ± 0.96^a^	17.82 ± 3.26^a^
	100 N	55.66*	29.10 ± 2.40^a^	16.64 ± 7.47^a^
^15^NH_4_^+^ (atom% ^15^N)	50 N	0.36	2.38 ± 0.76^a^	1.84 ± 0.56^a^
	100 N	0.36	2.71 ± 0.37^a^	3.05 ± 0.36^a^
*a*D	50 N	0.44 ± 0.01 ^a^	0.45 ± 0.01 ^a^	0.47 ± 0.01 ^a^
	100 N	0.47 ± 0.04^a^	0.48 ± 0.06 ^a^	0.49 ± 0.07 ^a^

Means denoted by a different lowercase letter indicate significant differences (P < 0.05).

^*^NO_3_^−^ levels and enrichment for day 0 were calculated by mass balance from the amount and enrichment of the labelled fertilizer added to the soil.

The ^15^N enrichment of the NO_3_^−^ pool undergoing denitrification (*a*D) was slightly lower than the theoretical ^15^N enrichment calculated from the addition of labelled fertilizer at 60% atom ^15^N-NO_3_^−^ to the existing soil mineral N pool (1.92 kg NO_3_^−^ N ha^−1^) at natural abundance (Table 2). There was no significant difference in *a*D between the two treatments and over time with average values of 0.48 and 0.46 for the 100 N and 50 N treatment, respectively.

## Discussion

### Performance of the novel Field-IRMS

The custom build Field-IRMS, a modified continuous flow IRMS coupled to a fully automated chamber system, enabled direct field measurements of N_2_ and N_2_O fluxes from^15^N labelled soil in real time at a sub-daily resolution. To our knowledge, this is the first time that automated real-time measurements of N_2_ and N_2_O from fertilized cropping systems are reported.

The most significant equipment modification was the sample preparation unit where we used a zeolite-based N_2_O trapping system and included automated sampling loops to transfer the air samples from the field chambers into the helium carrier flow of the TGP. The new Field-IRMS allows for simultaneous analysis of concentration and isotopic signature of N_2_ and N_2_O in the sample air with high analytical precision. The in batch standard deviation of atmospheric samples was used to define the instruments precision: For N_2_, the standard deviation of ^29^R was 4.70 * 10^−07^, which is within the reported precision of other IRMS^12,16^, while ^30^R at 1.41 * 10^−06^, was at the upper end of reported values^17^.

This precision of the IRMS translates to a detection limit of 53.9 g ha^−1^ day^−1^ and 0.25 g ha^−1^ day^−1^ for N_2_ and N_2_O emissions, respectively. These detection limits are comparable to or better than those reported by studies using the ^15^N gas flux method in fertilized cropping systems (62 to 380 g N ha^−1^ day^−1^ for N_2_ and 1.0 to 1.7 g N ha^−1^ day^−1^ for N_2_O)^18–20^; but higher than those reported by a recent study that adapted the ^15^N gas flux method for application in unfertilized land use systems (1.0 g N ha^−1^ day^−1^ for N_2_ and 0.05 * 10^−3^ g N ha^−1^ day^−1^ for N_2_O)^21^. However, it needs to be noted that the MDL of the ^15^N gas flux method not only depends on the sensitivity of the IRMS and can potentially be lowered by (1) using a higher ^15^N enrichment of the applied fertilizer (2) decreasing the chamber volume and (3) increasing the closure time of the static chamber. All these parameters need to be adjusted according to the experimental set-up, in particular the expected N_2_ and N_2_O emission range.

To ensure high-quality measurements are provided, our automated chambers were designed using state of the art quality protocols^22,23^. We chose to use relatively large chambers (basal area 0.25 m^2^, height 0.15 m), to reduce the error associated with environmental variables such as temperature, the N_2_O diffusion gradient within the soil and potential gas leaks into the chamber^24^. Furthermore, the larger chambers account better for spatial variation, known to be one of the great challenges in measuring denitrification in the field^25^, but require rather large amounts of costly ^15^N fertilizer.

Field studies using the ^15^N gas flux method in fertilized cropping systems typically only take two gas samples (t_0_ and t_1_) from the chamber over a 1 to 3 h closure time^19,26,27^, but in unfertilized systems closure times up to 20 h have been reported^21,28^. Long closure times have been shown to lead to an underestimation of denitrification rates due to decreasing concentration gradients between soil and chamber atmosphere, causing lowering of surface fluxes with increasing chamber deployment time and ideally, closure times should not exceed one hour^29^. This means that for field measurements closure times should be minimised according to the expected N_2_ and N_2_O emission range but must be long enough so that sensitivity is not compromised. Our systems offers the advantage that four samples are taken over a closure period of 201 min which a) allows to check for the linearity of the evolved N_2_ and N_2_O gases over the deployment time of each individual chamber, b) increases the precision of the flux determination and c) enables a better quality control of the calculated flux including the detection of outliers.

Another key advantage of the Field-IRMS is that it eliminates any errors associated with the manual extraction, transport, storage and injection of gas samples and accounts for diurnal variations of the measured N gas fluxes. The limited amount of studies that reported N_2_ fluxes from fertilized cropping systems typically used a sampling regime for N_2_ measurements ranging from daily to weekly measurements, which cannot fully account for the high temporal variation of denitrification. Consequently, the use of an automated system will significantly improve estimates of denitrification from fertilized cropping systems. Such data is urgently needed for the identification of sustainable cropping practices that reduce GHG emissions and N pollution from agricultural systems while increasing food security under the auspices of climate change.

However, the accuracy of the ^15^N gas flux method needs to be revisited since recent research shows that field surface fluxes of ^15^N-labelled N_2_ and N_2_O emitted from ^15^N-labelled soil NO_3_^−^ can severely underestimate denitrification due to subsoil flux and accumulation in pore space^29^, suggesting that denitrification in fertilised cropping systems could even be more important than previously thought. Ideally, future studies on denitrifcation using the ^15^N-gas flux method can be combined with an estimation of subsoil fluxes.

### Field experiment

Over the 7-day field campaign, significant N_2_ and N_2_O fluxes could be observed in both fertilizer treatments with 100% of the N_2_O measurements and 93% of the N_2_ measurements showing a significant linear increase in the chamber headspace, demonstrating the suitability of the new Field-IRMS to continuously measure N_2_ and N_2_O emissions in fertilized cropping systems. Denitrification was identified as a major loss pathway of fertilizer N with total denitrification losses (N_2_ + N_2_O) of 2.10 ± 0.46 kg ha^−1^ and 6.12 ± 0.99 kg ha^−1^ over the seven-day period for the 50 N and 100 N treatments. These high N losses are in the range of previously reported values from sugarcane cropping systems in Australia^27,30^. The only other study that used the ^15^N gas flux method in a sugarcane field reported total denitrification losses ranging from 7.6 to 9.2 kg ha^−1^ over nine days after the application of 160 kg N ha^−1^ K^15^NO_3_ (98.5 atom %)^27^. These results highlight that sub-tropical sugarcane systems in Australia can be a hotspot for soil denitrification where high denitrification losses can be expected in particular, since the high denitrification events occurred after 50 mm and 25 mm irrigation, respectively. Such rainfall events are characteristic for the humid subtropical summers in the study region and generally occur numerous times during the sugarcane growing season.

Fertilization and irrigation triggered an initial pulse of N_2_ and N_2_O emissions. High WFPS and NO_3_^−^ availability together with soluble C from the sugarcane trash created conditions favouring denitrification, resulting in emissions of up to 1.25 kg N ha^−1^ day^−1^ of N_2_ and N_2_O combined. Following this first irrigation N_2_ and N_2_O emissions decreased over the first three days as the soil dried, while the N_2_:N_2_O ratio shifted towards N_2_ in the 50 N treatment. A second irrigation on the night of the third day resulted in a significant increase of N_2_ while there was only a minor effect on N_2_O emissions. Such a burst of N_2_ and N_2_O emissions following irrigation and fertilization has been observed in other cropping systems and is typically caused by O_2_ depletion due to increased WFPS^26^, and further amplified by increased N substrate availability and the formation of anaerobic microsites due to enhanced microbial activity and subsequent O_2_ consumption^31,32^. The increase in the N_2_/N_2_ + N_2_O product ratio over time reflects increasing anaerobic conditions in the soil matrix along with enhanced N_2_O reductase activity, causing a shift towards complete denitrification to N_2_. The lower N_2_/(N_2_ + N_2_O) product ratio in the 100 N treatment can probably be attributed to higher soil NO_3_^−^ concentrations^33^ (Table 2), inhibiting N_2_O reductase activity due to the competitive effect of NO_3_^−^ and N_2_O as electron acceptors during denitrification.

Total denitrification losses were 3 times higher in the 100 N treatment than in the 50 N treatment, while N_2_O emissions were 5 times higher, which indicates that under the observed conditions of high moisture, temperature and C, denitrification was mainly limited by the NO_3_^−^ availability and suggests a non-linear trend of increasing denitrification rates as N inputs increase to exceed crop needs, similar to what has been observed for N_2_O emissions in fertilized cropping systems^34^.

Denitrification was dominated by N_2_ emissions, with an N_2_/(N_2_ + N_2_O) product ratio of 0.93 and 0.90 in the 50 N and 100 N treatment, respectively. These ratios are within the range of reported product ratios from other field studies that used the ^15^N gas flux method in fertilized cropping systems^19,26^, and agree well with the average product ratio of 0.85 ± 0.061 for fertilized agricultural soils by a recent meta-analysis that summarized available datasets where N_2_ emissions have been either measured by ^15^N-labelling approaches or with the gas-flow helium incubation method^3,35^. However, it needs to be noted that the magnitude and the N_2_/(N_2_ + N_2_O) product ratio of denitrification is known to vary substantially depending on soil conditions and is primarily influenced by the soil nitrate content, the availability of easily degradable C substrates, soil moisture, soil oxygen content, the genetic potential for N_2_O reduction and soil pH^17^,^36–39^. Consequently, longer-term measurements from different sugarcane sites are ideally required to come up with robust estimates of N_2_/(N_2_ + N_2_O) product ratios and total denitrification losses for sugarcane cropping systems.

Another problematic aspect of the ^15^N gas flux method is the determination of the ^15^N enrichment of the source pool (NO_3_^−^). We used the isotopic signatures of ^45^N_2_O:^44^N_2_O and ^46^N_2_O:^44^N_2_O in the headspace gas to calculate the ^15^N enrichment of the NO_3_^−^ pool undergoing denitrification *a*D. While there was no statistically significant difference between the ^15^NO_3_^−^ pool undergoing denitrification derived from ^15^N_2_O (*a*D) and from ^15^N_2_ (XN^15^) (Fig. S2), XN^15^ was consistently lower than *a*D. This effect is in agreement with other studies and has been attributed to a non-homogeneity of tracer dilution as a result of small scale heterogeneity of nitrification resulting in a stronger dilution of the NO_3_^−^ pool in oxic and lower dilution in anoxic microsites producing N_2_ and N_2_O^19,40^. But *a*D showed a much lower variation between replicates which suggests that the use of *aD* is under these conditions likely to be more reliable than XN^15^, since d, the fraction of N_2_O in the chamber headspace derived from denitrification is usually higher than the one for N_2_, as N_2_O is a trace gas, supporting the use of *a*D to calculate N_2_ fluxes^11^.

On the first sampling day after application of ^15^N fertilizer *a*D was 0.44 and 0.47 for the 50 N and 100 N treatment, respectively. This was lower than the theoretical value of 0.55 calculated by mass balance from the soil NO_3_^−^ content before fertilizer application and the amount and enrichment of the labelled fertilizer added to soil, indicating increasing nitrification or heterogeneity in the initial NO_3_^−^ distribution, and thus a higher contribution of non-fertilizer NO_3_^−^ to the denitrifying NO_3_^−^ pool. There was no significant change in *a*D over the course of the seven-day experiment, while measurements of the NO_3_^−^ pool enrichment determined by the diffusion method following KCl extraction on day 4 and 8 of the experiment showed significantly lower values and a declining trend over time. The results of the diffusions show a dilution of the soil NO_3_^−^ pools, most likely from nitrification of unlabelled NH_4_^+^. However, this did not change the enrichment of the active denitrifying pool, which indicates a spatial separation of denitrification and nitrification, occurring in different micro-sites within the soil matrix. It has been shown that direct measurement of ^15^N-NO_3_^−^ using extraction and diffusion does not reflect the enrichment of the active NO_3_^−^ pool undergoing denitrification, and the use of these data in denitrification rate calculations has been shown to result in unrealistically high N_2_ flux rates^20,41^.

The automated sampling cycle of the Field-IRMS provides robust estimates of Δ^29^R, Δ^30^R and *a*D, allowing to (a) test key assumptions of the ^15^N gas flux method and (b) to calculate N_2_ fluxes based on 4 point measurements. This approach represents a significant improvement compared to two point measurements and increases the precision of the flux determination. There is however still room for improvement regarding the sensitivity of the IRMS and the time needed per sample. Further improvements of the TGP and the sampling cycle including the integration of reference gas pulses injected directly into the IRMS and an adaption of the closure time in high emitting agro-ecosystems could further increase the temporal resolution of the Field-IRMS. Another promising approach could be to use this system with a reduced N_2_ atmosphere in the chamber headspace. This method has recently been used in field and lab studies and shown that it can significantly reduce the detection limit for N_2_ fluxes^41,42^. However, it requires a rather complex set-up involving flushing of the headspace and the sub-soil with an artificial gas mixture that needs to be coupled to the automated sampling system. Improving and combining these novel approaches to measure N_2_ could significantly improve our understanding of denitrification in fertilized and even natural agroecosystems.

## Supplementary information

Supporting Info
