# Supplementary material for: Mobile continuous-flow isotope-ratio mass spectrometer system for automated measurements of N2 and N2O fluxes in fertilized cropping systems
Source: Sci Rep. 2019 Jul 31;9:11097. doi: 10.1038/s41598-019-47451-7 (PMC6668390; doi:10.1038/s41598-019-47451-7)
Supplement: Supplementary file 1 — Supporting Info [file 41598_2019_47451_MOESM1_ESM.docx]

**SUPPORTING INFORMATION**

**Manuscript title:**

**Mobile continuous-flow isotope-ratio mass spectrometer system for automated measurements of N_2_ and N_2_O fluxes in fertilized cropping systems**

**Authors:**

Daniel I. Warner^1^, Clemens Scheer^1,2^, Johannes Friedl^1^, David W. Rowlings^1^, Christian Brunk^1^ and Peter R. Grace^1^

^1^ Institute of Future Environments, Queensland University of Technology, Brisbane, QLD 4000 Australia,

^2^ Institute for Meteorology and Climate Research, Atmospheric Environmental Research (IMK-IFU) Karlsruhe Institute of Technology (KIT) Garmisch-Partenkirchen, Germany.

^*^corresponding authors: clemens.scheer@kit.edu

johannes.friedl@qut.edu.au

Supporting information consists of 10 pages, 2 tables, and 2 figures.

# Automated sampling cycle

The N_2_ and N_2_O fluxes were measured using a fully automated sampling cycle during which four gas samples were taken sequentially from each chamber over the closure period. During each of the sampling runs nine blocks of reference standards were analysed which consisted of three ambient air and one 1 ppm N_2_O standard, injected from a certified calibration gas cylinder (SupaGas, Beenleigh, QLD, Australia). The ambient air standards were used to provide an isotopic reference for the N_2_ measurements and the N_2_O calibration standard was used to provide a reference for the N_2_O measurements. These reference blocks were run at the beginning and end of the runs and between every four chamber measurements. To analyse one gas sample with the IRMS for ^14/15^N_2_O and ^14/15^N_2_ concentrations took 20.10 min. Consequently, every 20.17 min a new sample or reference gas could be analysed. To optimise the sample timing a set of two chambers (i.e. chamber 1 & 2, chamber 3 & 4, chamber 5 & 6, chamber 7 & 8) was closed at the same time and sampled sequentially. Air samples are taken sequentially from each closed chamber followed by a reference block between the 2^nd^ and 3^rd^ measurements. In total each chamber was sampled four times over a 201.67 min period with a total closure time of 221.83 min (Table S1). Following the end of this sample cycle the chambers opened, another reference block was measured and the next set of two chambers were closed and the next sampling cycle started. This cycle was then repeated until all eight chambers had been sampled four times. With this sampling cycle it took a total of 22.85 hours to sample each chamber four times, leaving 1.25 hours to retune the IRMS and perform any necessary maintenance,

Table S1: Timing of sampling cycle for the first two chambers, all following chambers follow the same sample timing

| Sample number | Chamber 1 | Chamber 2 |
| --- | --- | --- |
| 1st | 0.00 min | 20.17 min |
| 2nd | 40.33 min | 60.50 min |
|  | Reference block | |
| 3rd | 161.33 min | 181.50 min |
| 4th | 201.67 min | 221.83 min |

# Flux calculations

Di-nitrogen and the converted N_2_O (N_2_+N_2_O) are measured as N_2_ in the Field-IRMS, while N_2_O is measured directly as N_2_O. The isotopologues of N_2_ (^14^N^14^N, ^14^N^15^N and ^15^N^15^N) and N_2_O (^14^N^14^N^16^O, ^14^N^15^N^16^O and ^15^N^15^N^16^O) were determined for each sample. For N_2_O, the ion currents (I) at the mass to charge ratio (*m/z)* 44, 45 and 46 enabled the he calculation of the molecular ratios ^45^R (^45^I/^44^I) and ^46^R (^46^I/^44^I).

## N_2_O concentration and fluxes

The 1 ppm N_2_O reference standard was used to calculate the absolute concentration of the N_2_O (ppm) in each sample. This was done according to calculations provided by Stevens, Laughlin et al. (1993). This method uses the isotopic ratios of all the possible N_2_O species, ^45^R, ^46^R, ^47^R and ^48^R, however a triple collector IRMS can only provide measured ratios for ^45^R and ^46^R. The ratios for ^47^R and ^48^R are provided by:

^47^R = (^15^R)^2^ * ^17^R + 2 * ^15^R * ^18^R (1)

^48^R = ^18^R * (^15^R)^2^ (2)

^17^R (^17^O/^16^O) and ^18^R (^18^O/^16^O) are assumed to be at natural abundance, therefore:

^17^R = 0.00037795 and

^18^R = 0.002079

Due to the non-random distribution of ^15^N in N_2_O the ^15^R needs to be calculated with information from both the measured ^45^R and ^46^R as follows:

${}^{15}NAt\%=100*( {}^{45}R+2*{}^{46}R-{}^{17}R-2*{}^{18}R)/(2+2*{}^{45}R+2*{}^{46}R)$ (3)

^15^R = (^15^N At% / 100) / (1 – ^15^N At% / 100) (4)

The absolute concentration of the N_2_O was then calculated using the concentration of N_2_O in the 1 ppm reference gas, assuming a linear respond of the IRMS to concentrations between 0 and 1 ppm. This was done by calculating an IRMS sensitivity factor (K^N2O^) based on the ^44^I of the reference gas:

K^N2O^ = ^44^I_r_/^44^M_r_ (5)

Where ^44^I_r_ = the mass 44 ion signal from the reference gas and ^44^M_r_ is given by using the theoretical calculations of the N_2_O ratios in the reference standard:

^44^M_r_ = M/(1 + ^45^R + ^46^R + ^47^R + ^48^R) (6)

Where M is the concentration of the reference gas

The concentration of the N_2_O in the chamber samples (M_s_) is then given by:

M_s_ = ^44^I_r_ * (1 + ^45^R_s_ + ^46^R_s_ + ^47^R_s_ + ^48^R_s_)/K^N2O^ (7)

Subscript (s) denotes sample and subscript (r) denotes reference.

The N_2_O fluxes were then calculated based on the slope of the linear increases in absolute N_2_O concentration (ppm) over the closure time of the chambers, which was corrected for temperate, pressure and the ratio of chamber volume to surface area with the following formula.

(8)

$$F_{N2O}=\frac{b\cdot V_{CH}\cdot MW\cdot60\cdot{10}^{6}}{A_{CH}\cdot{MV}_{corr}\cdot{10}^{9}}$$

Where F_N2O_ is the hourly flux of N_2_O (ug m^2^ h^-1^), b is the N_2_O concentration (ppb min^-1^), V_CH_ is the volume of the headspace within the chamber (m^3^), MW is the molecular weight of the N-N_2_O in this case 28, 60 converts hours to minutes, 10^6^ converts µg to g. A_CH_ is the surface area of the chamber (m^2^), MV_corr_ is the temperate and pressure correction for molecular volume and 10^9^ converts ppb to µL m^-3^. The N_2_O flux was then converted and expressed as N_2_O-N kg ha^-1^ day^-1^.

## Fluxes of N_2_

For N_2_, the ion currents at *m/z* 28, 29 and 30 enabled ^29^R (^29^I/^28^I) and ^30^R (^30^I/^28^I) to be calculated, with differences between ambient and enriched atmospheres expressed as Δ^29^R and Δ^30^R. The ^15^N enrichment of the soil NO_3_^-^ pool undergoing denitrification (^15^X_N_) is calculated following the equations of Mulvaney (1984) as

|  | $\mathrm{XN}^{15}=\frac{2\left( \frac{\Delta^{30}R}{\Delta^{29}R} \right)}{\left( 1+2\left( \frac{\Delta^{30}R}{\Delta^{29}R} \right) \right)}$ | (9) |
| --- | --- | --- |

It should be noted that equations 9 & 10 can lead to bias if XN^15^ < 0.2. This problem can be avoided by the use of equations from Spott, Russow et al. (2006). However in this study XN^15^ was consistently high (> 0.4) and therefore the equations of Mulvaney (1984) were used.

The fraction of N_2_ in the chamber headspace derived from denitrification d is then estimated as

| $d=\frac{\Delta^{30}R}{\left( \mathrm{XN}^{15} \right)^{2}}$ | (10) |
| --- | --- |

Assuming that N_2_O produced via denitrification and N_2_ originate from the same NO_3_^-^ pool of the same ^15^N enrichment, the ^15^N enrichment of the NO_3_^-^ pool undergoing denitrification can be estimated based on the isotopologues of N_2_O (Stevens and Laughlin 2001). As N_2_O is produced along the NH_4_^+^ oxidation pathway or denitrification, the sources of N_2_O are divided into the fraction of N_2_O (*d*’D) derived from the denitrifying pool of enrichment *a*D and the fraction *d*’N=(1- *d*’D) derived from the pool or pools of natural abundance following the equations given by Arah (1997):

*a*D = (x_s_ – a_s_ * a_a_) / (a_s_ – a_a_) (11)

where a_a_ is the ^15^N atom fraction of the background atmosphere, a_s_ is the ^15^N atom fraction of the headspace sample and x_s_ is the ^30^N_2_ molecular fraction of the sample (all referring to the of the N_2_O component). The fraction of N_2_O derived from denitrification is given by:

*d*’D = (a_s_ – a_a_)^2^ / (x_s_- a_s_ * a_a_+a^2^_A_) (12)

Multiplying N_2_O fluxes by *d*’D gives then N_2_O emissions derived from denitrification.

The fraction of N_2_ derived from denitrification *d* is then calculated using *a*D in equation 10 instead of ^15^X_N_ from equation 9_,_ this substitution assumes that N_2_ and N_2_O are formed from the same, uniformly labelled source pool. The comparison of *a*D and ^15^X_N_ suggests that both N_2_O and N_2_ were produced from a single source pool (Figure S2), supporting the use of *a*D to calculate N_2_ fluxes (Stevens and Laughlin 2001). The use of *aD* is under these conditions i.e. simultaneous emission of N_2_ and N_2_O from a single source pool likely to be more reliable than XN^15^, since d, the fraction of N_2_O in the chamber headspace derived from denitrification is usually higher than the one for N_2_, as N_2_O is a trace gas. The amount of N_2_ inside the chamber headspace multiplied by *d* then gives the amount of N_2_ evolved using equation 13.

| Evolved N (g) = N_2[chamber]_ * d | (13) |
| --- | --- |

Where N_2[chamber]_ is the total N_2_ in the chamber corrected for air pressure and temperature:

N_2[chamber]_ (g) = (V_ch_ * N_2con_) * temp ^o^C / N_2ideal_) * MW (14)

Where V_ch_ is the volume of chamber (mL), N_2con_ is the concentration of N_2_ in atmosphere, N_2ideal_ is one mole of gas at standard pressure and temperature and MW is the molecular mass of N_2_.

The flux of measured N_2_+N_2_O (F_N2+N2O_) was then calculated by creating a slope of the increase in evolved N_2_ of the 4 sample measurements.

$F_{N2+N2O}(g {ha}^{-1}{day}^{-1})=((b\cdot1000000) / A_{ch}\cdot60*10000)*0.24$ (15)

The use of a slope to calculate the increase in F_N2+N2O_ allowed us to use the R^2^ of the slope to reject fluxes where the R_2_ was less than 0.8 (Figure S1). As F_N2+N2O_ comprises both N_2_ and converted N_2_O, the flux of N_2_ was calculated as the difference between N_2_+N_2_O and N_2_O fluxes.

Figure S1: The accumulation of mass 30 (^15^N^15^N) (a) and mass 29 (^14^N^15^N) (b) N_2_, total evolved N_2_ (c) and N_2_O (d) in the chamber headspace over the 201 min measuring time for chamber 8 at day 7 of the experiment.

Figure S2: Estimates of the enrichment of the soil NO_3_^-^ pool undergoing denitrification: Comparison of ^15^X_N_ (calculated based on isotopologues of N_2_), *a*D (calculated based on isotopologues of N_2_O), and the ^15^N enrichment of extractable NO_3_^-^ (1:5 w:V; 2M KCl extract).

Table S2: Physiochemical properties in the soil profile of the experimental site.

| Depth (cm) | Clay (%) | Silt (%) | Sand (%) | TOC (mg/g) | TN (mg/g) | pH water | EC (dS/m) |
| --- | --- | --- | --- | --- | --- | --- | --- |
| 0–10 | 8 | 7 | 85 | 10.0 | 0.68 | 5.9 | 0.054 |
| 10–20 | 8 | 7 | 85 | 9.6 | 0.64 | 6.0 | 0.050 |
| 20–30 | 9 | 7 | 84 | 8.0 | 0.54 | 6.0 | 0.040 |
| 30–60 | 15 | 6 | 79 | 4.0 | 0.34 | 6.0 | 0.037 |
| 60–100 | 25 | 6 | 69 | 1.6 | 0.23 | 5.7 | 0.033 |

# Experimental site

The field experiment was carried out at a commercial sugarcane farm system near Bundaberg, QLD Australia (24^o^57’53”S, 152^o^20’0”E). The long-term (1959–2012) annual mean temperature an the study site is 21.5 ^o^C (Bundaberg Aero Station, the Bureau of Meteorology, Australia), with the lowest monthly mean temperature in July (16.1 ^o^C) and the highest in January (25.8 ^o^C). Mean annual rainfall is 1027 mm, with ca. 56% of rainfall received from December to March.  The soil is a redoxic Hydrosol (Isbell, 2002) with loamy sand in the 0–30 cm layer, underlain by sandy loam at about 30–60 cm depth and sandy clay loam at about 60–100 cm depth, full description in (Table S2).

# References

Arah, J. R. M. (1997). "Apportioning nitrous oxide fluxes between nitrification and denitrification using gas-phase mass spectrometry." Soil Biology and Biochemistry **29**(8): 1295-1299.

Mulvaney, R. L. (1984). "Determination of 15N-Labeled Dinitrogen and Nitrous Oxide with Triple-Collector Mass Spectrometers1." Soil Science Society of America Journal **48**(3): 690-692.

Spott, O., R. Russow, B. Apelt and C. F. Stange (2006). "A 15N-aided artificial atmosphere gas flow technique for online determination of soil N2 release using the zeolite Köstrolith SX6®." Rapid Communications in Mass Spectrometry **20**(22): 3267-3274.

Stevens, R. J. and R. J. Laughlin (2001). "Lowering the detection limit for dinitrogen using the enrichment of nitrous oxide." Soil Biology & Biochemistry **33**(9): 1287-1289.

Stevens, R. J., R. J. Laughlin, G. J. Atkins and S. J. Prosser (1993). "Automated Determination of Nitrogen-15-Labeled Dinitrogen and Nitrous Oxide by Mass Spectrometry." Soil Sci. Soc. Am. J. **57**(4): 981-988.
